# Aflrpn4 Represents a Promising Target for Mitigating *Aspergillus flavus* Growth and Aflatoxin Contamination

**DOI:** 10.3390/toxins18070284

**Published:** 2026-06-29

**Authors:** Xingsai Liu, Yanli Xin, Kashif Iqbal Sahibzada, Xiujia Zhang, Cunjian Tu, Shan Wei, Yuansen Hu, Yangyong Lv

**Affiliations:** 1 Food Laboratory of Zhongyuan, Luohe 462044, China; lxs2810@stu.haut.edu.cn (X.L.);; 2 College of Biological Engineering, Henan University of Technology, Zhengzhou 450001, China; 3 Department of Health Professional Technologies, Faculty of Allied Health Sciences, The University of Lahore, Lahore 54570, Pakistan

**Keywords:** *Aspergillus flavus*, aflatoxin B1, Aflrpn4, oxidative stress, grain storage

## Abstract

*Aspergillus flavus* and its primary secondary metabolite, aflatoxin B1, pose a persistent threat to global food security and public health, highlighting the need to identify novel molecular targets for the development of highly specific fungicides. In this study, the transcription factor Aflrpn4 was investigated by constructing gene deletion and complementation strains to elucidate its regulatory mechanisms in controlling the growth, development, and pathogenicity of *A. flavus*. Phenotypic analysis revealed that, compared with the wild-type and complemented strains, loss of *Aflrpn4* severely restricted radial colony growth, reduced conidial yield, and caused structural defects in conidiophores. Furthermore, AFB1 content was reduced by 52% compared with the wild-type. In storage simulation assays using peanut and maize kernels, the *ΔAflrpn4* strain exhibited significantly compromised colonization capacity, reduced biomass, and lower AFB1 accumulation. Under aflatoxin-inducing YES culture conditions, deletion of *Aflrpn4* was associated with significant downregulation of key pathway-specific regulatory and structural genes, including *aflR*, *aflS*, and *aflP*. Furthermore, under osmotic stress induced by 1.2 M NaCl and KCl, the growth inhibition rates of the *ΔAflrpn4* strain reached 70% and 59%, respectively, and cell membrane integrity was severely compromised. Loss of *Aflrpn4* also disrupted intracellular redox homeostasis, characterized by a significant decrease in superoxide dismutase activity, compensatory increases in catalase and peroxidase activities, and substantial accumulation of reactive oxygen species. Collectively, these findings demonstrate that Aflrpn4 acts as a pivotal regulator coordinating vegetative growth, asexual development, stress adaptation, and aflatoxin biosynthesis in *A. flavus*. Consequently, Aflrpn4 represents a promising molecular target for developing targeted interventions to control *A. flavus* and aflatoxin contamination during grain storage.

## 1. Introduction

*Aspergillus flavus* and the associated aflatoxins stand out as contaminants for many agricultural products, posing a persistent threat to global food security and public health. The Food and Agriculture Organization (FAO) estimates that about 25% of all food crops are contaminated by mycotoxins each year, with a strong correlation between aflatoxin exposure and human hepatocellular carcinoma [1]. Synthetic chemical fungicides, widely used to manage *A. flavus* contamination, have raised environmental concerns, led to the accumulation of chemical residues in food chains, and contributed to the rapid development of isolates resistant to chemical fungicides [2]. Therefore, there is an urgent need to develop new, highly specific, eco-friendly antifungal drugs [3,4]. Achieving this will require detailed knowledge of the molecular networks that control fungal growth, environmental adaptation, and secondary metabolism to identify new druggable targets [5,6].

The fungal cell membrane and its regulatory pathways are potential targets for antifungal drug development. The transcription factor Rpn4 plays a conserved role in maintaining cellular homeostasis under stress in yeast and various human pathogenic fungi. For example, CgRpn4 regulates membrane permeability and ergosterol biosynthesis in *Candida glabrata*, affecting susceptibility and resistance to azole antifungals [7]. Similarly, the *Rpn4* gene in *Candida auris* has been shown to confer fluconazole resistance by transcriptionally upregulating multidrug efflux pumps [8]. In *Candida albicans*, *Rpn4* is essential for ergosterol synthesis, and its disruption impairs lipid metabolism and membrane integrity, eliminating tolerance to fluconazole [9]. Moreover, studies in *Saccharomyces cerevisiae* have shown that deletion of *RPN4* is associated with the induction of endoplasmic reticulum (ER) stress, disruption of lipid homeostasis, and decreased cell viability under adverse environmental conditions [10,11]. Although important insights have been gained in yeasts and clinical pathogens, the biological roles and regulatory mechanisms of *Rpn4* homologues in filamentous phytopathogens, particularly *A. flavus*, remain poorly understood.

Since *Rpn4* is also involved in stress adaptation and maintaining cell membrane homeostasis, we hypothesized that the *A. flavus* homologue, *Aflrpn4*, plays a critical role in coordinating cell membrane integrity, environmental stress responses, and aflatoxin biosynthesis. To test this, we introduced a deletion mutation in the *Aflrpn4* gene (*ΔAflrpn4*) and generated a complemented strain (*ΔAflrpn4-com*). Using these mutants, this study systematically elucidates the specific effects of *Aflrpn4* deletion on vegetative growth, asexual sporulation, pathogenicity of *A. flavus* during peanut and maize storage, tolerance to osmotic stress, cell membrane permeability, and intracellular redox homeostasis. It also comprehensively evaluates the regulatory effects of *Aflrpn4* on aflatoxin biosynthesis. By addressing these questions, this study provides novel insights into the regulatory network of *A. flavus* and establishes *Aflrpn4* as a promising molecular target for the development of targeted control strategies in grain storage.

## 2. Results

### 2.1. Sequence Analysis of Aflrpn4 and Construction of Mutant Strains

**Figure 1:**
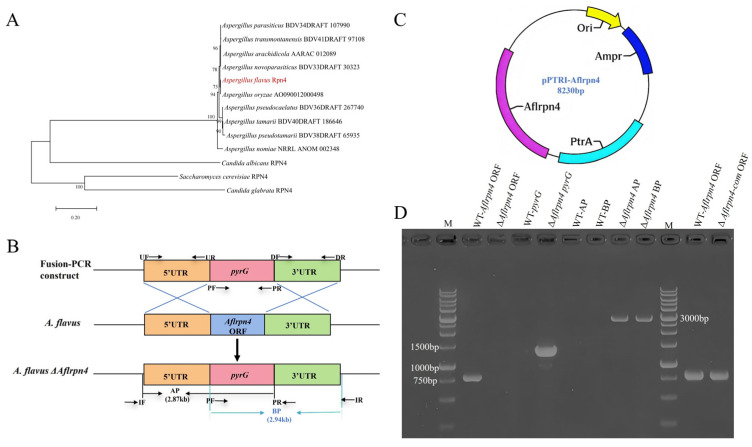
Phylogenetic characterization of *Aflrpn4* and generation of mutant strains in *A. flavus*: (**A**) Phylogenetic tree showing the evolutionary relationships of *Aflrpn4* with its homologues across various species. (**B**) Schematic representation of the homologous recombination strategy used for *Aflrpn4* gene deletion. (**C**) Plasmid map of the complementation vector pPTRI-*Aflrpn4* used to construct the complemented strain. (**D**) PCR-based validation of the mutant strains.

The *S. cerevisiae Rpn4* sequence (YDL020C) was used as a query to identify its homologue in the *A. flavus* genome database, designated as *Aflrpn4*. Phylogenetic analysis of *Aflrpn4* using MEGA 6.0 showed high conservation of *Aflrpn4* among filamentous fungi, with 32.8% identity to *S. cerevisiae Rpn4* (Figure 1A). In addition, diagnostic PCR was performed to confirm the successful gene deletion mutant, in which the target gene was replaced with the *pyrG* selectable marker, and the complemented strain, which was constructed by introducing the pPTR1-*Aflrpn4* plasmid into the deletion mutant (Figure 1B–D).

### 2.2. Aflrpn4 Deletion Attenuates Colonization and Aflatoxin Production on Crop Seeds

**Figure 2:**
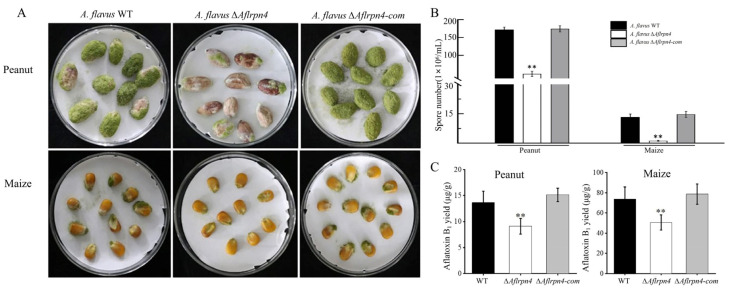
Impact of *Aflrpn4* deletion on the virulence of *A. flavus* during peanut and maize infection: (**A**) Representative colonization phenotypes of peanut and maize kernels inoculated with the WT, *ΔAflrpn4*, and *ΔAflrpn4-com* strains. (**B**) Quantitative analysis of conidial production by the indicated strains on infected crop substrates. (**C**) Aflatoxin B1 accumulation in peanut and maize kernels post-infection. Data are presented as mean ± SD. Asterisks (**) indicate highly significant differences (*p* < 0.01) compared to the WT strain.

Peanut and maize seeds underwent infection assays to assess the impact of *Aflrpn4* on pathogenicity during storage. After five days of incubation, the wild-type and complemented strains heavily colonized the seeds, producing dense mycelial networks and abundant green conidia (Figure 2A). In contrast, the *ΔAflrpn4* mutant exhibited significantly reduced colonization, with a marked decrease in conidial production on both peanut and maize surfaces (Figure 2B). Furthermore, HPLC analysis revealed lower levels of AFB1 in seeds infected with the *ΔAflrpn4* mutant compared to the wild-type and complemented strains (Figure 2C), demonstrating the importance of *Aflrpn4* in the colonization and toxigenic potential of *A. flavus* on agricultural commodities.

### 2.3. Aflrpn4 Is Required for Vegetative Growth and Asexual Development

**Figure 3:**
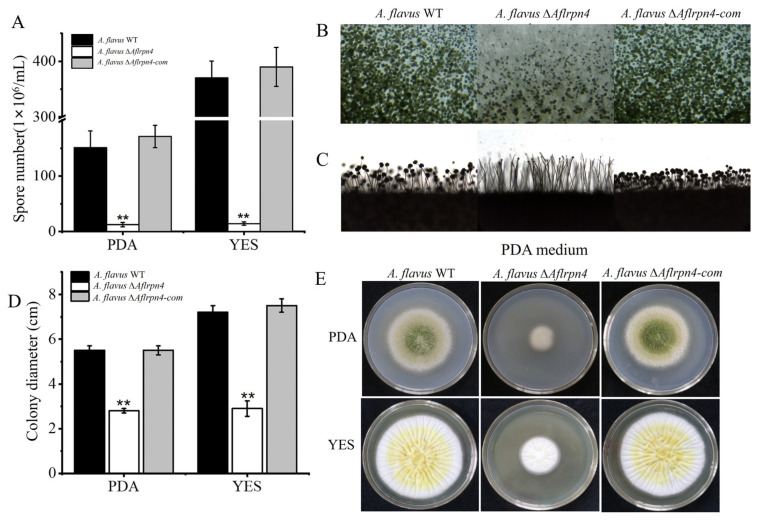
Influence of *Aflrpn4* deletion on asexual development and vegetative growth of *A. flavus*: (**A**) Quantitative analysis of conidial production in the WT, *ΔAflrpn4*, and *ΔAflrpn4-com* strains after 5 days of cultivation on PDA and YES media. (**B**) Conidial head morphology of the indicated strains on PDA medium. (**C**) Microscopic observation of conidiophore structures. (**D**) Statistical comparison of colony diameters among the three strains on PDA and YES media. (**E**) Representative colony phenotypes on PDA and YES agar plates. Data are presented as mean ± SD. Asterisks (**) indicate highly significant differences (*p* < 0.01).

To evaluate the role of *Aflrpn4* in asexual development, we quantified conidial production and examined conidiophore morphology in the WT, *ΔAflrpn4*, and *ΔAflrpn4-com* strains. The *ΔAflrpn4* mutant showed a drastic reduction in conidial yield on both PDA and YES media compared to the WT and complemented strains (Figure 3A,B). Microscopic observation revealed that, while the WT and *ΔAflrpn4-com* strains developed dense, typical conidiophores with globose conidial heads, the conidiophores of the *ΔAflrpn4* mutant were sparse, elongated, and barren, resembling withered grass due to a failure in proper conidial head maturation (Figure 3C). These findings highlight the essential role of *Aflrpn4* in normal conidiogenesis in *A. flavus*. Given the severe defect in asexual reproduction, we next investigated the effects of *Aflrpn4* deletion on vegetative growth by preparing standardized conidial suspensions of the three strains and point-inoculating them onto PDA and YES agar plates, followed by five days of incubation. The radial growth of the *ΔAflrpn4* mutant was significantly restricted, with significantly smaller colony diameters as compared to the WT (Figure 3D,E). Notably, the complemented strain (*ΔAflrpn4-com*) completely restored the radial growth phenotype to wild-type levels. Overall, these findings suggest that *Aflrpn4* plays a critical role in regulating both vegetative growth and asexual development in *A. flavus*.

### 2.4. Loss of Aflrpn4 Downregulates Aflatoxin Biosynthetic Genes and Inhibits Toxin Synthesis

**Figure 4:**
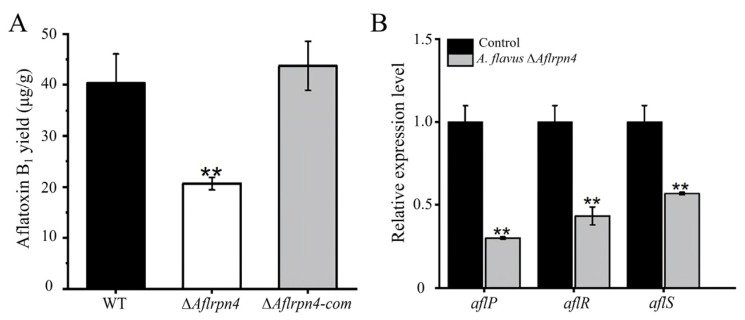
Impact of *Aflrpn4* deletion on aflatoxin B1 biosynthesis and expression of pathway-specific genes: (**A**) High-performance liquid chromatography (HPLC) quantification of AFB1 yields. (**B**) Relative expression levels of AFB1 biosynthetic genes (*aflP*, *aflR*, and *aflS*) determined by qRT-PCR. Data represent the mean ± SD of three independent replicates. Asterisks (**) indicate highly significant differences (*p* < 0.01).

Toxin production was further quantified using HPLC, which confirmed reduced production of toxin (52% reduction in AFB1) in *Aflrpn4* compared to the WT (Figure 4A). To investigate the molecular mechanism underlying this hypotoxigenic phenotype, qRT-PCR was performed to assess the transcription levels of key genes within the AFB1 gene cluster, including the structural gene *aflP* and the pathway-specific regulatory genes *aflR* and *aflS*. The expression levels of all three genes were significantly downregulated in the *ΔAflrpn4* mutant, showing a substantial decrease compared to the WT control (Figure 4B). These findings indicate that *Aflrpn4* positively regulates the transcription of key aflatoxin biosynthetic genes under YES culture conditions. The observed reduction in *aflR*, *aflS*, and *aflP* expression provides a potential molecular explanation for the decreased aflatoxin production phenotype associated with *Aflrpn4* deletion.

### 2.5. Aflrpn4 Modulates Osmotic Stress Tolerance and Cell Membrane Integrity

**Figure 5:**
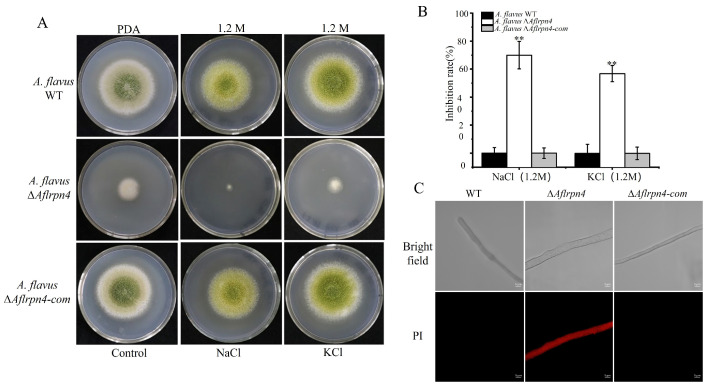
Role of *Aflrpn4* in hyperosmotic stress tolerance and cell membrane integrity in *A. flavus*: (**A**) Colony morphology of the WT, *ΔAflrpn4*, and *ΔAflrpn4-com* strains cultivated on PDA (control) or PDA supplemented with 1.2 M NaCl or 1.2 M KCl for 5 days. (**B**) Radial growth inhibition rates of the indicated strains under hyperosmotic stress. (**C**) Fluorescence microscopy of hyphae stained with propidium iodide (PI) to assess cell membrane integrity (scale bars = 5 μm). Data are presented as mean ± SD of three independent replicates. Asterisks (**) indicate highly significant differences (*p* < 0.01) compared to the WT.

Osmotic and cell membrane stressors were used to assess the role of *Aflrpn4* in environmental stress adaptation. Osmotic stress was induced by 1.2 M NaCl or KCl, which severely restricted the growth of the *ΔAflrpn4* mutant, with inhibition rates reaching 70% and 59%, respectively. These rates were significantly higher than those observed in the WT and complemented strains (Figure 5A,B). PI staining was conducted to evaluate the association between sensitivity and cell membrane defects. The results showed intense red fluorescence in the mycelia of the *ΔAflrpn4* mutant, whereas the WT and complemented strains exhibited negligible staining (Figure 5C), indicating that *Aflrpn4* is essential for maintaining cell membrane integrity and adapting to osmotic stress.

### 2.6. Deletion of Aflrpn4 Triggers Intracellular ROS Accumulation and Alters Antioxidant Enzyme Activities

**Figure 6:**
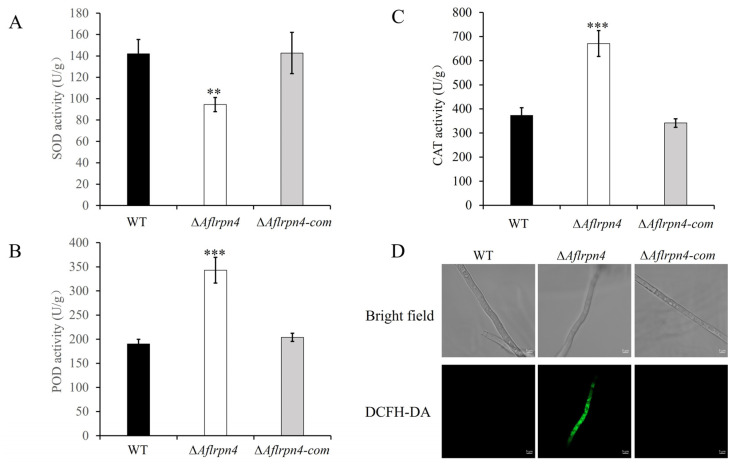
Effects of *Aflrpn4* deletion on intracellular ROS accumulation and antioxidant enzyme activities in *A. flavus*: (**A**–**C**) Intracellular activities of superoxide dismutase (SOD) (**A**), peroxidase (POD) (**B**), and catalase (CAT) (**C**) in the WT, *ΔAflrpn4*, and *ΔAflrpn4-com* strains. (**D**) Representative bright-field and fluorescence micrographs of hyphae stained with the ROS-sensitive probe DCFH-DA (scale bars = 5 μm). Data are expressed as mean ± SD from three independent replicates. Asterisks indicate statistically significant differences compared with the WT (** *p* < 0.01, *** *p* < 0.001).

Intracellular reactive oxygen species (ROS) levels were monitored using the fluorogenic probe 2′,7′-dichlorodihydrofluorescein diacetate (DCFH-DA) to evaluate the impact of *Aflrpn4* deletion on the cellular redox status of *A. flavus*. Additionally, activities of antioxidant enzymes were measured. Biochemical assays revealed a significant reduction in SOD activity in the mutant (*ΔAflrpn4*) compared to the WT and *ΔAflrpn4-com* strains (Figure 6A). In contrast, peroxidase (POD; Figure 6B) and catalase (CAT; Figure 6C) activities were significantly elevated in the mutant strain. With these enzymatic alterations, fluorescence microscopy of DCFH-DA-stained hyphae showed a marked increase in green fluorescence intensity in the *ΔAflrpn4* mutant compared to the control strains, confirming substantial intracellular ROS accumulation (Figure 6D). These findings indicate that loss of *Aflrpn4* disrupts cellular redox homeostasis, triggering oxidative stress and compensatory modulation of the enzymatic antioxidant defense system in *A. flavus*.

## 3. Discussion

*A. flavus* and its associated aflatoxins, which are potential contaminants of grains, food products, and animal feed, pose serious threats to public health and cause substantial economic losses globally. Consequently, there is a need to develop effective strategies to control *A. flavus* growth and reduce mycotoxin production in agricultural commodities. Targeting specific regulatory factors and structural genes in the biosynthetic pathway of aflatoxins has proven to be a highly effective approach. For example, Wang et al. [4] identified small-molecule inhibitors that target the AflG structure to suppress toxin synthesis, while Han et al. [3] demonstrated that fusing the AflR DNA-binding motif with an antimicrobial peptide significantly reduced both the minimum inhibitory concentration (MIC) and aflatoxin production. However, designing highly specific and eco-friendly antifungal agents requires the identification of novel druggable molecular targets that affect both fungal development and toxigenesis. In this study, *Aflrpn4* is characterized as a key regulator that coordinates vegetative growth, asexual development, stress adaptation, and aflatoxin biosynthesis in *A. flavus*, establishing it as a potential target for post-harvest grain protection.

Fungal growth kinetics, host colonization efficiency, and sporulation capacity are important factors in the virulence and competitiveness of *A. flavus* during grain storage [12,13]. Our phenotypic characterization revealed that these pathogenic characteristics were severely affected by the deletion of *Aflrpn4*, resulting in restricted radial growth, defective conidiophore development, and a substantial reduction in AFB1 synthesis (approximately 52% in liquid culture). Additionally, the *ΔAflrpn4* mutant showed a dramatic decrease in colonization ability under simulated peanut and maize storage conditions, with biomass accumulation reduced by more than 65% compared to the WT strain. These developmental and toxigenic defects parallel those observed upon deletion of established global or pathway-specific regulators in *A. flavus*, such as the pathway-specific transcription factors AflR [14] and AflS [15], or the velvet family protein VeA [16]. Based on the similarities in phenotypes, the gene *Aflrpn4* occupies a high-level hierarchical position in the regulatory network governing fungal development and secondary metabolism, making it a promising target for small-molecule inhibitors aimed at disrupting *A. flavus* colonization and toxigenic potential at the source.

Fungi must be able to adapt to changes in environmental osmolarity and maintain the integrity of their cell membranes to survive and carry out normal metabolic processes [17]. Our findings show that the growth rate of the *ΔAflrpn4* mutant is highly sensitive to hyperosmotic conditions, with 70% and 59% growth inhibition under 1.2 M NaCl and KCl stress, respectively. This increased sensitivity was accompanied by a marked rise in cell membrane permeability, as indicated by intense propidium iodide (PI) staining in the mutant hyphae. In *A. flavus*, osmotic stress responses are generally regulated by the SakA/Hog1 mitogen-activated protein kinase (MAPK) pathway and small GTPases such as Rac [18,19]. Deletion of sakA/Hog1 results in increased sensitivity to osmotic stabilizers (e.g., sorbitol), while simultaneously upregulating host colonization and aflatoxin biosynthesis [19]. Conversely, the deletion of Rac leads to impaired stress tolerance and toxigenesis [18]. The phenotype of the *ΔAflrpn4* mutant is very similar to that of the Rac deletion, indicating that *Aflrpn4* may function in parallel with or downstream of pathways that regulate both stress adaptation and virulence. This is further supported by studies on other antifungal agents and bacterial metabolites of *Achromobacter xylosoxidans,* which disrupt the cell membrane and lead to apoptosis in *A. flavus* [20], highlighting membrane-associated regulatory pathways as highly vulnerable therapeutic targets.

It is well established that intracellular redox homeostasis and reactive oxygen species (ROS) dynamics are closely linked to development and secondary metabolism in fungi [21]. Previous studies have shown that deletion of oxidative stress regulators or exposure to natural compounds such as curcumin can trigger an intracellular ROS burst, leading to downregulation of aflatoxin biosynthetic genes [22,23]. In the *ΔAflrpn4* mutant, we observed that the enzymatic antioxidant defense system was disrupted, with a significant decline in SOD activity and compensatory increases in CAT and POD activities. Despite these compensatory responses, the mutant exhibited a high level of intracellular ROS. This oxidative imbalance correlated with dramatic transcriptional downregulation of the pathway-specific regulators *aflR* and *aflS*, as well as the key structural gene *aflP*. Our findings suggest that *Aflrpn4* is a critical node in the regulation of ROS homeostasis and secondary metabolism, likely by integrating cellular stress signals with the transcriptional activation of the aflatoxin gene cluster.

One limitation of this study is that transcriptional analyses were performed in liquid YES medium rather than directly on infected kernels. Because substrate composition can affect aflatoxin biosynthesis and gene expression, the transcriptional responses observed under YES culture conditions may not fully reflect those occurring during kernel colonization. However, the consistent reduction in aflatoxin accumulation observed on both peanut and maize kernels, along with the downregulation of *aflR*, *aflS*, and *aflP* under aflatoxin-inducing conditions, supports a positive regulatory role for *Aflrpn4* in aflatoxin biosynthesis. Future studies using transcriptomic or targeted gene expression analyses during kernel infection will further clarify the substrate-dependent regulatory functions of *Aflrpn4*.

## 4. Conclusions

**Figure 7:**
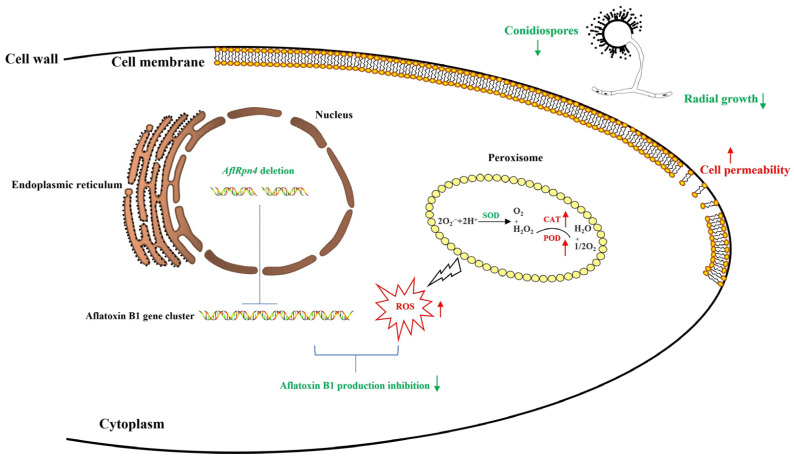
Proposed schematic model illustrating the regulatory network of *Aflrpn4* in *A. flavus*. The red and green arrows indicate the upregulation and downregulation of the relevant items or genes respectively.

In conclusion, deletion of *Aflrpn4* triggers a cascade of cellular defects, including impaired vegetative growth, abnormal conidiophore development, compromised cell membrane integrity, and disrupted redox homeostasis. This results in the accumulation of intracellular ROS and downregulation of key biosynthetic genes (*aflR*, *aflS*, and *aflP*), ultimately leading to a significant decrease in aflatoxin B1 production (Figure 7). Given its dual role in regulating both fungal viability and mycotoxin synthesis, *Aflrpn4* is a highly promising molecular target for developing targeted control strategies to mitigate *A. flavus* contamination during grain storage.

## 5. Materials and Methods

### 5.1. Plasmids, Strains, and Culture Conditions

ANIp7 obtained from FGSC (https://www.fgsc.net/) was used to amplify the pyrG selectable marker. pMD20T (TAKAR, Dalian, China) was used to construct the *Aflrpn4* knockout vector, while pPTRI (TAKAR, Dalian, China) was used to generate the *Aflrpn4* complementation vector. *A. flavus* TXZ21.3 [24] was used as the parental strain for generating the *Aflrpn4* gene deletion mutant. The transformation protocol for *A. flavus* followed the method described by Lv et al. [25]. All strains, including the wild-type (WT), the *Aflrpn4* deletion mutant (*ΔAflrpn4*), and the complemented strain (*ΔAflrpn4-com*), were routinely maintained on potato dextrose agar (PDA) or yeast extract-sucrose (YES) medium at 30 °C.

### 5.2. Construction of ΔAflrpn4 and ΔAflrpn4-com Strains

To construct the *Aflrpn4* gene deletion cassette, homologous recombination was used. The upstream and downstream flanking regions of *Aflrpn4* (AFLA_017640) were amplified from *A. flavus* genomic DNA using specific primer pairs (Aflrpn4-up-F/R and Aflrpn4-down-F/R). Plasmid ANIp7 was used to amplify the pyrG selectable marker gene. These three fragments were cloned into the pMD20 vector using a multi-fragment ligation kit (Vazyme, Nanjing, China) to generate the recombinant deletion plasmid. The deletion cassette was then amplified and transformed into protoplasts of *A. flavus* TXZ21.3.

For complementation, the full-length *Aflrpn4* gene, including its native promoter, open reading frame, and terminator, was amplified and cloned into the pPTR1 vector. The resulting plasmid, pPTR1-*Aflrpn4*, was transformed into *ΔAflrpn4* protoplasts, and transformants were selected on media containing pyrithiamine. Successful gene deletion and complementation were verified by PCR analysis.

### 5.3. Virulence and Colonization Assays on Peanut and Maize Seeds

To evaluate the role of *Aflrpn4* in pathogenicity during storage, healthy, uniform peanut and maize seeds were sterilized and inoculated with spore suspensions (10^6^ spores/mL) following the method of Lv et al. [25]. After incubation at 30 °C for 5 days, conidial production and AFB1 accumulation on the infected peanut and maize kernels were quantified according to a previous method [26].

### 5.4. Assessment of Vegetative Growth and Conidiation

A 1 μL aliquot of spore suspension (10^6^ spores/mL) from the WT, *ΔAflrpn4*, and *ΔAflrpn4-com* strains was inoculated onto PDA and YES agar plates and incubated at 30 °C for 5 days (three replicates per strain). Colony diameters were measured daily using the cross-intersection method. For conidial quantification, the colonies were washed with sterile water, and the spores were suspended and counted using a hemocytometer. Conidiophore morphology was examined by cultivating the strains on thin agar blocks placed on sterile glass slides at 30 °C for 12 h, followed by microscopic observation.

### 5.5. Quantification of Aflatoxin B1 and Gene Expression Analysis

For mechanistic analyses of aflatoxin biosynthesis, strains were cultured in liquid YES medium, a commonly used aflatoxin-inducing medium that provides uniform growth conditions and facilitates reproducible RNA extraction and gene expression analysis [27]. Kernel infection assays were used to assess virulence and aflatoxin accumulation under storage-related conditions, while YES medium was used to investigate the transcriptional regulation of aflatoxin biosynthetic genes under controlled laboratory conditions [28]. Spore suspensions (10^6^ spores/mL) were inoculated into liquid YES medium and cultured at 30 °C with shaking at 200 rpm for 3 days. Mycelia were harvested, immediately frozen in liquid nitrogen, and ground for RNA extraction. Total RNA was reverse-transcribed into cDNA [29], and quantitative real-time PCR (qRT-PCR) was performed to determine the expression levels of key aflatoxin biosynthetic genes (*aflP*, *aflR*, and *aflS*) using the 2^−ΔΔCT^ method [30], with the actin gene as the internal reference.

The culture filtrate (10 mL) was extracted with an equal volume of chloroform for AFB1 quantification. The organic phase was isolated, dried, and reconstituted in methanol. High-performance liquid chromatography (HPLC) coupled with a post-column photochemical derivatizer (Shimadzu, Kyoto, Japan) and a fluorescence detector (Shimadzu, RF-20A) was used to determine AFB1 levels. Separation was achieved on a C18 column (4.6 × 250 mm, 5 μm) with a mobile phase of methanol–water (6:4, *v*/*v*) at a flow rate of 1 mL/min. For each group of samples, the excitation wavelength of the fluorescence detector was set at 360 nm and the emission wavelength at 440 nm to determine AFB1 content. Under these conditions, the retention time of AFB1 is approximately 4.4 min.

### 5.6. Osmotic Stress Tolerance and Cell Membrane Permeability Assays

Osmotic and cell membrane stress sensitivity were evaluated by inoculating 1 μL of spore suspension (10^6^ spores/mL) onto PDA plates supplemented with NaCl or KCl (0.8, 1.0, or 1.2 M). After 5 days of incubation at 30 °C, growth inhibition rates were calculated by comparing colony diameters.

To assess cell membrane integrity, the propidium iodide (PI) staining method was used. Mycelia were cultured in DPY liquid medium for 3 days, then harvested, washed with phosphate-buffered saline (PBS), and incubated in the dark with PI staining solution at 30 °C for 30 min. After washing, the samples were visualized under a confocal laser scanning microscope LSM900 (Carl Zeiss, Jena, Germany).

### 5.7. Quantification of Antioxidant Enzyme Activities and Intracellular Reactive Oxygen Species (ROS)

The harvested mycelia were ground in liquid nitrogen to measure the activities of various antioxidant enzymes, including superoxide dismutase (SOD), catalase (CAT), and peroxidase (POD), using commercial assay kits (Solarbio, Beijing, China). The concentration of intracellular ROS was detected using the fluorescent probe 2′,7′-dichlorodihydrofluorescein diacetate (DCFH-DA). Green fluorescence intensity was measured in harvested mycelia incubated with DCFH-DA in the dark at 30 °C for 30 min, washed thoroughly with PBS, and imaged using a fluorescence microscope.

## Data Availability

The original contributions presented in this study are included in the article/Supplementary Materials. Further inquiries can be directed to the corresponding author.

## Supplementary Materials

The following supporting information can be downloaded at: https://www.mdpi.com/article/10.3390/toxins18070284/s1. Table S1. Primers used in this study.
