# Supplementary material for: Aflrpn4 Represents a Promising Target for Mitigating Aspergillus flavus Growth and Aflatoxin Contamination
Source: Toxins (Basel). 2026 Jun 29;18(7):284. doi: 10.3390/toxins18070284 (PMC13417813; doi:10.3390/toxins18070284)
Supplement: Supplementary file 1 [file toxins-18-00284-s001.zip › toxins-4396875-supplementary.pdf]

Supplementary Materials: *Aflrpn4* represents a promising target for mitigating *Aspergillus flavus* growth and aflatoxin contamination

Xingsai Liu, Yanli Xin, Kashif Iqbal Sahibzada, Xiujia Zhang, Cunjian Tu, Shan Wei, Yuansen Hu, and Yangyong Lv

Table S1. Primers used in this study

Primer Name	Primer sequences (5′-3′)
Aflrpn4-up-F	ctagaggatctactagtcatatggattCTTGATGCTGGGTAATCGTGAGTGA
Aflrpn4-up-R	tgcggcgcgttctcgaggaagttgcAGTGGATGGGGAAGTTCAGAGGATT
Aflrpn4-down-F	tgtgtaacggtattgactaaaagggTTTGAAACGCCTACGAAGCTGCA
Aflrpn4-down-R	tcgagctcggtacccggggatccgattTTTGATTCTTGCGGGCGAAGGATAC
Aflrpn4-com-F	ctagaggatctactagtcatatggattGACCGGTTGAGTCATGATCGCC
Aflrpn4-com-R	ggatcccgtaatcaattgcccCGCCTGACACCACAACCCATTC
Aflrpn4-ORF-F	TTCCAAGACCCCGTTTTCATCCAGG
Aflrpn4-ORF-R	AGGCCTCGTCAAGTTCCTCCGGTTT
pyrG-F	GCAACTTCCTCGAGAACGCGCCGCA
pyrG-R	CCCTTTTAGTCAATACCGTTACACA
aflP-F	ACGAAGCCACTGGTAGAGGAGATG
aflP-R	GTGAATGACGGCAGGCAGGT
aflR-F	GCGCATAACACGTACTCAAC
aflR-R	GGAGACGCTACTGCTACCAT
aflS-F	CGAGTCGCTCAGGCGCTCAA
aflS-R	GCTCAGACTGACCGCCGCTC
Actin-F	ACGGTGTCGTCACAAACTGG
Actin-R	CGGTTGGACTTAGGGTTGATAG
